# Medical Items

**Published:** 1913-03

**Authors:** 


### MEDICAL ITEMS

The many cases of nasal and naso-pharyngeal inflammation which are so prevalent at this season, call to mind the fact that Glyco-Thymoline is almost a specific in their treatment.

The cooling, soothing and slightly anodyne effect of this preparation on the dry and hot mucous surfaces is well known to the profession. In addition Glyco-Thymoline will by its exosmotic properties relieve the congested area of the inflammatory exudate and by stimulating the capillaries to renewed activity bring about a normal condition of the parts.

